# Redox Buffering Effects in Potentiometric Detection of DNA Using Thiol-Modified Gold Electrodes

**DOI:** 10.1021/acssensors.0c02700

**Published:** 2021-06-29

**Authors:** Xingxing Xu, Yingtao Yu, Qitao Hu, Si Chen, Leif Nyholm, Zhen Zhang

**Affiliations:** † Division of Solid-State Electronics, Department of Electrical Engineering, Ångström Laboratory, Uppsala University, P.O. Box 65, Uppsala SE-75103, Sweden; ‡ Department of Chemistry-Ångström Laboratory, Uppsala University, P.O. Box 538, SE-751 21 Uppsala, Sweden

**Keywords:** redox buffering effect, gold, field-effect transistor, potentiometric DNA detection, self-assembled monolayer

## Abstract

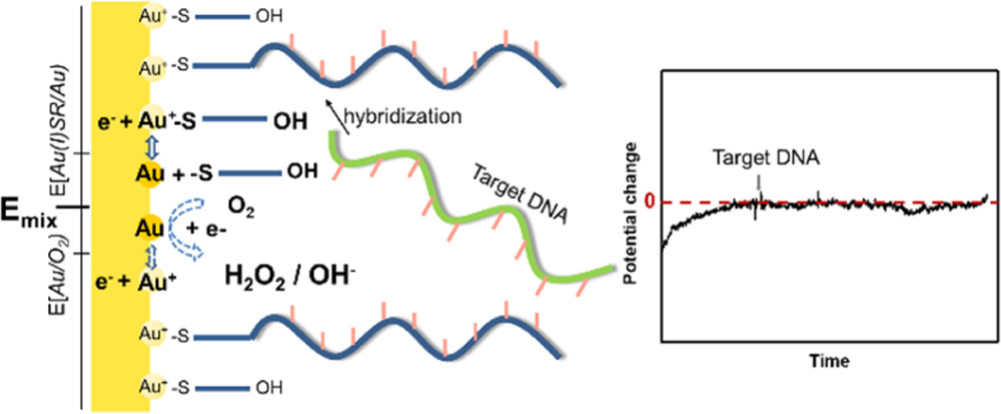

Label-free potentiometric detection of DNA molecules using a field-effect transistor (FET) with a gold gate offers an electrical sensing platform for rapid, straightforward, and inexpensive analyses of nucleic acid samples. To induce DNA hybridization on the FET sensor surface to enable potentiometric detection, probe DNA that is complementary to the target DNA has to be immobilized on the FET gate surface. A common method for probe DNA functionalization is based on thiol–gold chemistry, immobilizing thiol-modified probe DNA on a gold gate with thiol–gold bonds. A self-assembled monolayer (SAM), based on the same thiol–gold chemistry, is also needed to passivate the rest of the gold gate surface to prevent non-specific adsorption and to enable favorable steric configuration of the probe DNA. Herein, the applicability of such FET-based potentiometric DNA sensing was carefully investigated, using a silicon nanoribbon FET with a gold-sensing gate modified with thiol–gold chemistry. We discover that the potential of the gold-sensing electrode is determined by the mixed potential of the gold–thiol and gold–oxygen redox interactions. This mixed potential gives rise to a redox buffer effect which buffers the change in the surface charge induced by the DNA hybridization, thus suppressing the potentiometric signal. Analogous redox buffer effects may also be present for other types of potentiometric detections of biomarkers based on thiol–gold chemistry.

Due to the advantages including label-free detection, compatibility with large-scale production, and high speed, field-effect transistors (FETs) have been extensively explored for potentiometric determinations of pH, small ions, and biomolecules such as biomarkers.^1−4^ The DNA field-effect transistor (DNA-FET) is among the explorations of the applications of FET aiming at direct label-free electrical detection of DNA hybridization.^5,6,9^ A DNA-FET is usually made by immobilizing a probe DNA, that is complementary to the target DNA, on the surface of the FET gate. Theoretically, the target-probe DNA hybridization can introduce an additional negative charge on the gate, which could lead to a change in the surface potential and, hence, a change in the FET threshold voltage (Δ*V*_T_) and source-to-drain current (*I*_SD_).^1^ There are two common ways to functionalize the gate surface of the ISFET, that is, by immobilizing the probe DNA on a gate oxide surface using a linker^7,8,15^ or by immobilizing thiol-modified probe DNA on a gold metal gate,^9,10^ employing thiol–gold chemistry.^9,10^

However, there are many factors limiting the performance of a DNA-FET. These factors have resulted in large differences between the results presented in different reports.^5,7,11,12^ The limiting factors include the probe DNA coverage density (Γ_Probe_), the ionic strength of the sample, and the side reactions at the sensing surface.^11−14^ In our previous paper, we systematically studied the detection limit of a potentiometric DNA sensor induced by probe-target DNA hybridization on a gold-sensing surface and its dependence on Γ_Probe_ and the ionic strength, based on the results obtained with the surface plasmon resonance (SPR) technique.^15^ However, this estimation of the detection limit did not take the side reactions on the sensing surface into account and therefore merely indicates the maximum potentiometric signal generated by the DNA hybridization.

Side reactions at the sensor surface could introduce potential buffering effects which may suppress the potentiometric signal generated by the bound analyte. The most-studied side reaction is the pH buffering effect for oxide-sensing interfaces where the charge induced by the adsorption of the analyte can be buffered by proton exchange on the sensing surface. It has been reported that the high surface charge density of amphoteric SiO_2_, which results in high pH sensitivity, can significantly suppress the DNA-FET signal.^16^ The resulting potential change caused by the DNA hybridization can then be suppressed to as little as 4–5 mV.^16^ Researchers have likewise found that similar pH buffering effects can completely suppress the ISFET signal associated with protein detection.^17^ Proton interactions with gold oxide have also been found to decrease the ISFET signal for potentiometric detection of Ca^2+^.^18^

The influence of side reaction effects has rarely been studied for DNA sensors with thiol-modified gold-sensing surfaces. In this work, we carefully evaluate the possibilities of using potentiometric DNA detection employing a gold-gated silicon-nanoribbon FET (SiNRFET) sensor, using an optimized thiol-based surface DNA hybridization protocol.^15^ We find that the potential of the gold-sensing electrode is controlled by the mixed potential of the gold–thiol and gold–oxygen redox interactions. Their associated redox buffer capacities make it difficult to detect the potentiometric signal generated by the DNA hybridization.

## Materials and Methods

### Materials

6-Mercapto-1-hexanol (MCH) (HS(CH_2_)_6_OH, 97%), Tris(2-carboxyethyl)phosphine hydrochloride (TCEP), and sodium chloride (NaCl, 99.9%) were obtained from Sigma-Aldrich (Germany). The 10 mM Tris–EDTA solution [TE, 10 mM Tris, 1 mM ethylenediaminetetraacetic acid (EDTA), pH 8] and Tris buffer (10 mM Tris–HCl, pH 7.5) were obtained from ThermoFisher Scientific (Sweden). Ethanol (99.5%) was supplied by VWR (Sweden) whereas SU-8 2002 was obtained from MicroChem (USA). All chemicals, which were of analytical grade or better, were used as received. All aqueous solutions were prepared with ultrapure water with a resistivity higher than 18 MΩ·cm.

The oligonucleotides, which were purchased from Integrated DNA Technologies (Canada), had the following sequences: 5′-HO-(CH_2_)_6_–S–S–(CH_2_)_6_GCATTGGTCTACAAGTGAATCTCGA-3′ for the thiol-modified probe DNA and TCGAGATTCACTTGTAGACCAATGC for the target DNA. The oligonucleotides were hydrated in 10 mM TE buffer to yield a concentration of 100 μM, and aliquots were kept at −20 °C for long-term storage.

## Methods

### Fabrication of Gold-Gated SiNRFETs and Gold Electrodes

**Figure 1:**
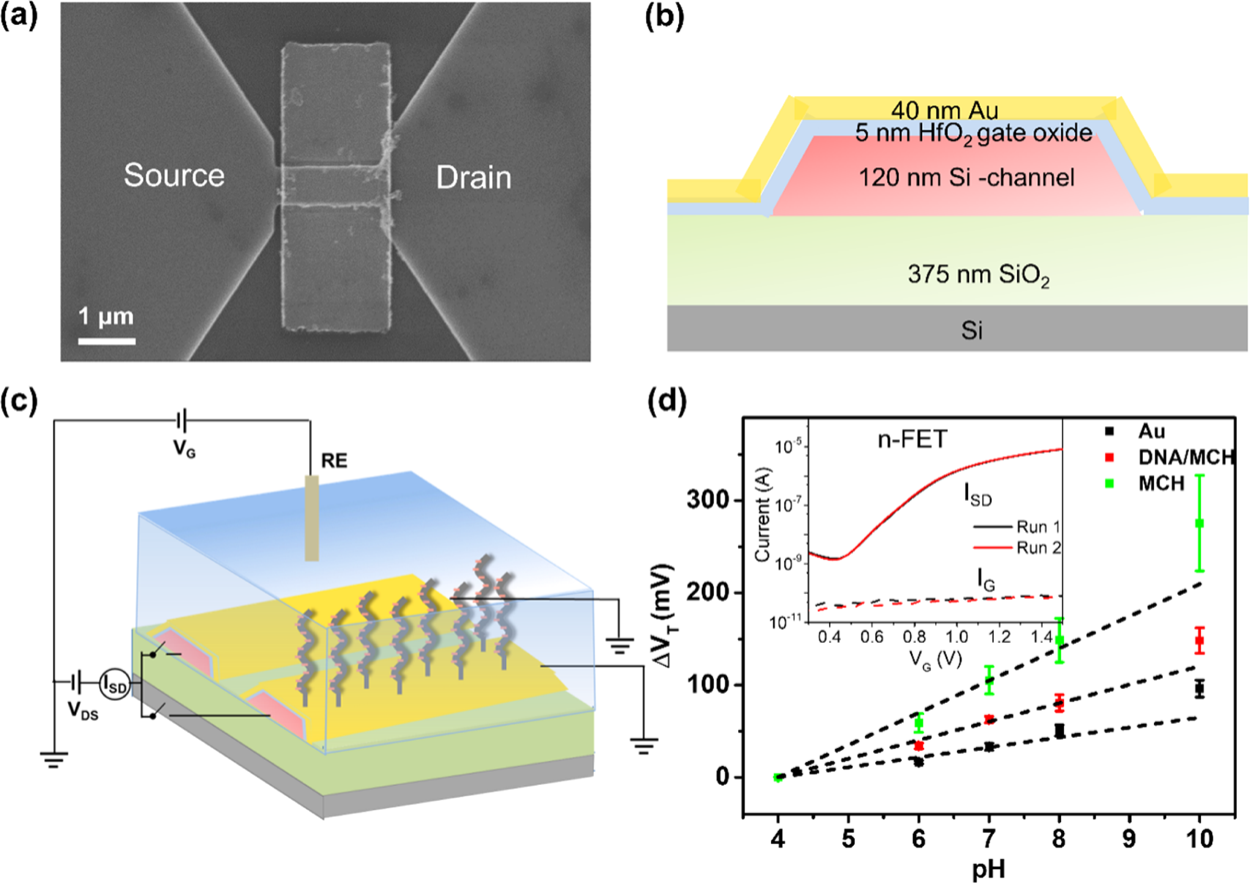
(a) Top-view SEM image of a SiNRFET with its channel region coated with gold. (b) Cross-section schematic of a gold-coated SiNRFET. (c) Sketch of the differential measurement setup. (d) pH sensitivities of the SiNRFETs with bare gold, MCH-modified gold, and DNA/MCH-modified gold as the sensing surfaces. The inset depicts the transfer curves of a DNA/MCH-modified gold SiNRFET recorded in Tris buffer containing 10 mM NaCl of two runs. As seen, the source-drain current (*I*_SD_) vs gate voltage (*V*_G_) curves and gate leakage current (*I*_G_)–V_G_ curves overlapped for these two different runs.

Potentiometric determinations of DNA were made using SiNRFETs with a gold-coated gate surface [see Figure 1a,b for the scanning electron microscopy (SEM) image and the cross-section schematic]. The SiNRFETs were fabricated on a silicon-on-insulator wafer with a 200 nm lightly p-type doped silicon layer and a 375 nm buried silicon dioxide layer using standard silicon process technology. The 200 nm lightly p-type doped silicon layer was first thinned down to 120 nm by thermal oxidation. Arsenic was then implanted into the source/drain (S/D) region (energy = 30 keV, dose = 5 × 10^15^/cm^2^) with the channel region protected by a photoresist during the implantation. Electron beam lithography was used to form a photoresist mask and reactive ion etching was then used to define the device structure. The resulting 24 nanoribbons were either 100 nm wide and 1 μm long or 500 nm wide and 2 μm long. A 5 nm thick Ni layer was evaporated and patterned on the n^+^ S/D region via lift-off. NiSi was subsequently formed in a rapid thermal process at 400 °C in N_2_ for 30 s. Afterward, a high-quality 5 nm HfO_2_ dielectric was grown by atomic layer deposition (R200 ALD unit, Picosun) at 170 °C to obtain a passivation layer in the electrolyte. The S/D contacts were metalized with 10 nm Ti and 100 nm Al via lift-off, after etching the HfO_2_ layer locally. A 40 nm-thick gold layer with 10 nm Ti as an adhesion layer was patterned on the silicon channel region, that is, the gate surface, also using a lift-off process (see Figure 1a for the top-view SEM image). The conformal sidewall coverage of gold was achieved with a two-step gold deposition process, with the chip tilted 60° clockwise and 60° anticlockwise during the first and the second deposition, respectively. Finally, forming gas annealing was performed in diluted H_2_ (5% H_2_ in N_2_, 400 °C, 30 min) to increase the interface quality of the Si channel and gate oxide.

The gold electrodes used in the open circuit potential (*E*_OC_) measurements were fabricated on an optically polished PYREX borosilicate glass (Präzisions Glas & Optik, Germany). The 100 nm thick thermally evaporated gold layer on 10 nm titanium was patterned by standard UV photolithography and lift-off processes. An SU-8 2002 photoresist was used to define the 0.00071 cm^2^ (diameter 0.3 mm) working electrode surface area and the 0.071 cm^2^ (diameter 3 mm) counter electrode area.

### Surface Modification

The surface modification process, which is described in the following, can also be found in our previous study.^15^ Prior to the surface modification, the SiNRFET chip was cleaned with O_2_ plasma (100 W) for 5 min and incubated in ethanol for 30 min to reduce the gold oxide. The 50 μM probe DNA was reduced with 50 mM TCEP for 1 h at room temperature and then diluted to 100 nM. The probe DNA was heated at 95 °C for 5 min and cooled on ice for 10 min prior to immobilization in order to linearize the DNA. Half of the SiNRFETs on the chip were then incubated in solutions containing probe DNA for 16 h at room temperature using a polydimethylsiloxane (PDMS, SYLGARD 184 Silicone Elastomer) container, while the other half was not exposed to this step. The latter SiNRFETs were hence used as control devices in the differential measurements. Afterward, the chip was rinsed with Tris buffer for 5 min, prior to incubation in 1 mM MCH in Tris buffer for 3 h to remove the non-specifically adsorbed probe DNA and to block the remaining gold surface area. Finally, the chip was washed with Tris buffer five times and with water five times.

### Potentiometric Measurement with the Gold-Gated SiNRFET

The potentiometric measurements were performed using an HP4155 semiconductor parameter analyzer. Up to 24 SiNRFETs can be measured simultaneously using a switch unit (Keithley 34970A). A schematic view of the measurement cell is shown in Figure 1c. The transfer curve (see the inset figure in Figure 1d for an example) was first measured. The *I*_SD_ was then monitored in real-time with a constant source-drain voltage (*V*_SD_) of 1 V and a constant *V*_G_ applied with respect to a Ag/AgCl/KCl reference electrode (leakage-free reference electrode, 1 mm in diameter, Warner Instruments, USA). The V_G_ during the measurement was in the subthreshold region. The *I*_SD_ was then converted to a *V*_T_ shift (Δ*V*_T_) based on the transfer curve.

A microfluidic system with Pump 11 Pico Plus Elite programmable syringe pumps (Harvard Apparatus) was used for the solution exchange during the potentiometric measurements. The employed microfluidic channel was fabricated using PDMS. The pH sensitivity measurements were conducted in pH buffer solutions made using Hydrion Chemvelope pH buffer powder (Micro Essential Laboratory, The USA). The components for each pH buffer were potassium bipthalate for the pH 4 buffer and a mixture of potassium phosphate (monobasic) and sodium phosphate (dibasic) for the pH 6, 7, and 8 buffers, as well as sodium carbonate for the pH 10 buffer. The potentiometric DNA detection measurements were performed in Tris buffer with different NaCl concentrations as described in the Results and Discussion section.

### Open Circuit Potential Measurements

The open circuit potential (E_OC_) measurements were conducted using a three-electrode cell and a VSP 300 (Bio-Logic, France) electrochemical workstation. These experiments were made with the abovementioned 0.00071 cm^2^ (diameter 0.3 mm) working electrodes and the 0.071 cm^2^ (diameter 3 mm) counter electrodes deposited on PYREX borosilicate glass pieces. The potential of the gold working electrode was measured versus the Ag/AgCl/sat. KCl reference electrode (Warner Instruments, USA).

## Results and Discussion

A top-view SEM image and the cross-section schematic of a gold-gated SiNRFET are shown in Figure 1a,b, respectively. A differential setup^4^ for potentiometric DNA detection was used to, as much as possible, eliminate the common signal drift caused by, for example, the reference electrode or non-specific interactions, during the measurements. The differential signals were obtained by subtracting the potential change of the control SiNRFETs from that of the sensing SiNRFETs. A schematic sketch of the measurement setup is shown in Figure 1c. During the measurements, the sensing SiNRFETs and the control SiNRFETs were biased using a common reference electrode and the *I*_SD_ of the sensing SiNRFETs and the control SiNRFETs were monitored simultaneously. The sensing SiNRFETs were functionalized with both probe DNA and MCH, while the control SiNRFETs were only modified with MCH (i.e., no probe DNA). As a result of the immobilization using a probe DNA concentration of 100 nM, Γ_Probe_ should have been in the range of 5.7 × 10^12^ to 8.2 × 10^12^ molecules/cm^2^ according to our previous work.^15^ A Γ_Probe_ value of 6.8 × 10^12^ molecules/cm^2^ was therefore assumed in this work. The SiNRFETs were n-type FETs as confirmed by the transfer curve in the inset of Figure 1d measured on a sensing SiNRFET. Moreover, the overlapped transfer curves from repeated measurements suggest that the SiNRFETs and the sensing interface were very stable under repeated liquid measurements. The FET V_T_ will shift in the positive direction to compensate the negative charge induced by the adsorption of negatively charged species and vice versa. This trend was confirmed by pH sensitivity measurements in the pH buffer solutions on the SiNRFET. As seen in Figure 1d, for all the samples, the *V*_T_ shift (Δ*V*_T_) was positive and increased when increasing the pH of the electrolyte from 4 to 10. Moreover, by linear fitting the measurement plots, the pH sensitivities of the bare gold, the MCH-modified gold (control samples), and the DNA/MCH-modified gold (sensing samples) were found to be 11 ± 2, 35 ± 3, and 20 ± 2 mV/pH, respectively. Here, the uncertainties depict the standard deviations calculated based on three independent measurements. The differences between the pH sensitivities of the MCH-modified and the DNA/MCH-modified gold surfaces, incidentally, also indicate that the immobilization of the probe DNA was successful.

In our previous paper,^15^ it was assumed that the total net surface charge induced by the hybridized target DNA (*Q*_h_) could charge the double layer capacitance (*C*_dl_) thus shifting the *V*_T_ of the DNA-FET. The estimated Δ*V*_T_ may then be calculated as
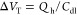
1

**Figure 2:**
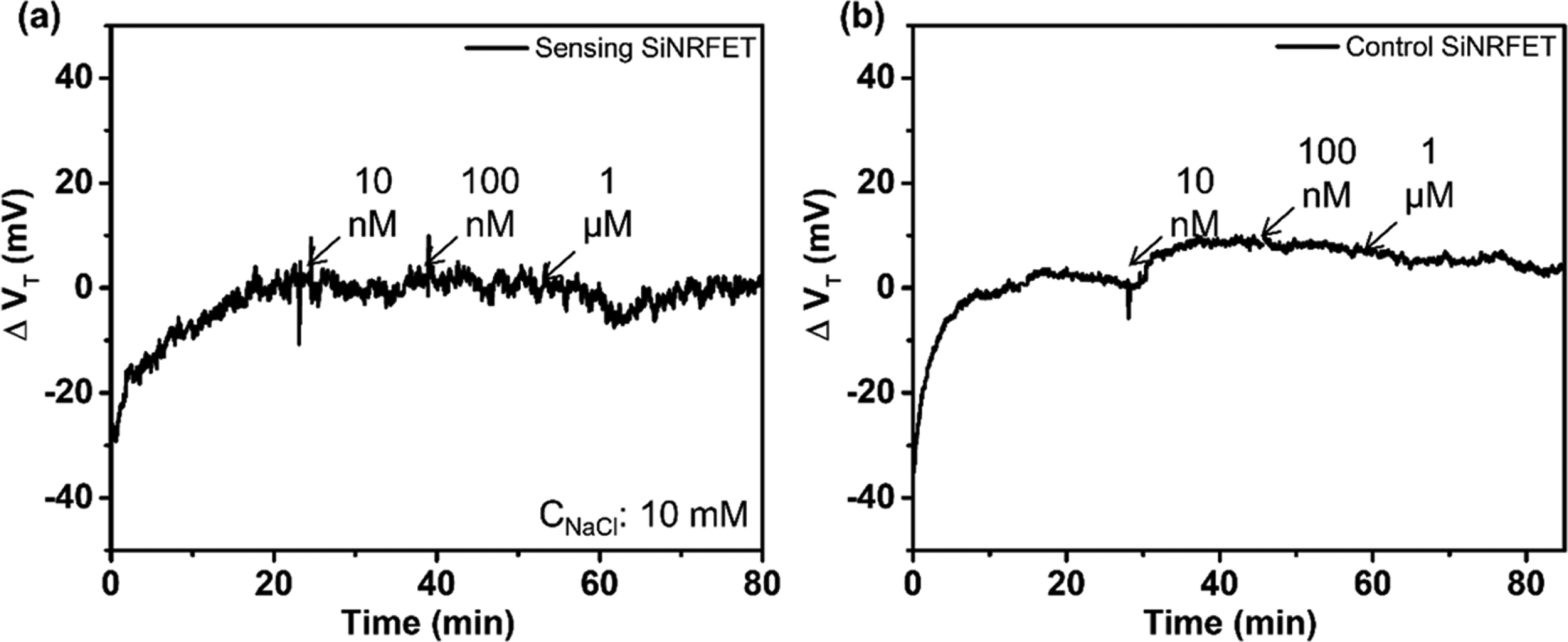
Real-time potentiometric detection of DNA in Tris buffer containing 10 mM NaCl using (a) the sensing SiNRFET and (b) the control SiNRFET. The injected target concentration was 10 nM, 100 nM, and 1000 nM, respectively. During each injection, the flow rate was initially set to 100 μL/min for 10 s to quickly replace the electrolyte in the microfluidic channel and then set to 5 μL/min for 15 min.

Here, *Q*_h_ would be determined by the hybridized target coverage density (Γ_Target_) and the number of DNA bases within the Debye length (λ_D_). A *C*_dl_ value of 4 μF/cm^2^ was used in this estimation. To enable the attainment of larger Δ*V*_T_ values, two methods, that is, the diluted buffer method increasing λ_D_ and an alternative method involving hybridization in a high ionic strength and measuring in low ionic strength buffer, were investigated. With the diluted buffer method, that is, potentiometric measurements in Tris buffers containing 1, 10, 100, and 1000 mM NaCl, the potentiometric signals were estimated and the maximum signal caused by target-probe hybridization was found to be as expected in Tris buffer containing 10 mM NaCl.^15^ Given a Γ_Probe_ value of 6.8 × 10^12^ molecules/cm^2^, the ionic strength in this buffer could enable three bases from one target DNA to be located in the λ_D_ and a Γ_Target_ value of around 3.7 × 10^11^ molecules/cm^2^, thus resulting a maximum detectable *Q*_h_ of around 1.8 × 10^–7^ C/cm^2^.^15^ Based on eq 1, the maximum potential change, in the absence of any side reaction, would then be around 44 mV. In this study, potentiometric detection of DNA using the gold-coated SiNRFETs was first performed in Tris buffer containing 10 mM NaCl (Figure 2) to compare the experimental results with the expected results stated above. As aforementioned, a positive Δ*V*_T_ should be expected after the target DNA injection since the hybridization of the target DNA with the surface probe DNA should introduce an additional negative charge on the gold surface. However, the real-time measurements using SiNRFETs showed that no potentiometric signal induced by this probe-target DNA hybridization could be registered. As seen in Figure 2a, once the Δ*V*_T_ had stabilized, 10, 100, and 1000 nM target DNA were successively injected in the same buffer where each injection lasted for 15 min. The injection of target DNA did, however, not generate a noticeable change in *V*_T_ even with the 1000 nM target concentration for the sensing SiNRFETs. Similar results were also found with the control SiNRFETs (Figure 2b). Although the measurements were repeated using two different sensing SiNRFETs, no noticeable change in Δ*V*_T_ caused by DNA hybridization could be registered for any of them (See Figure S1 in Supporting Information).

**Figure 3:**
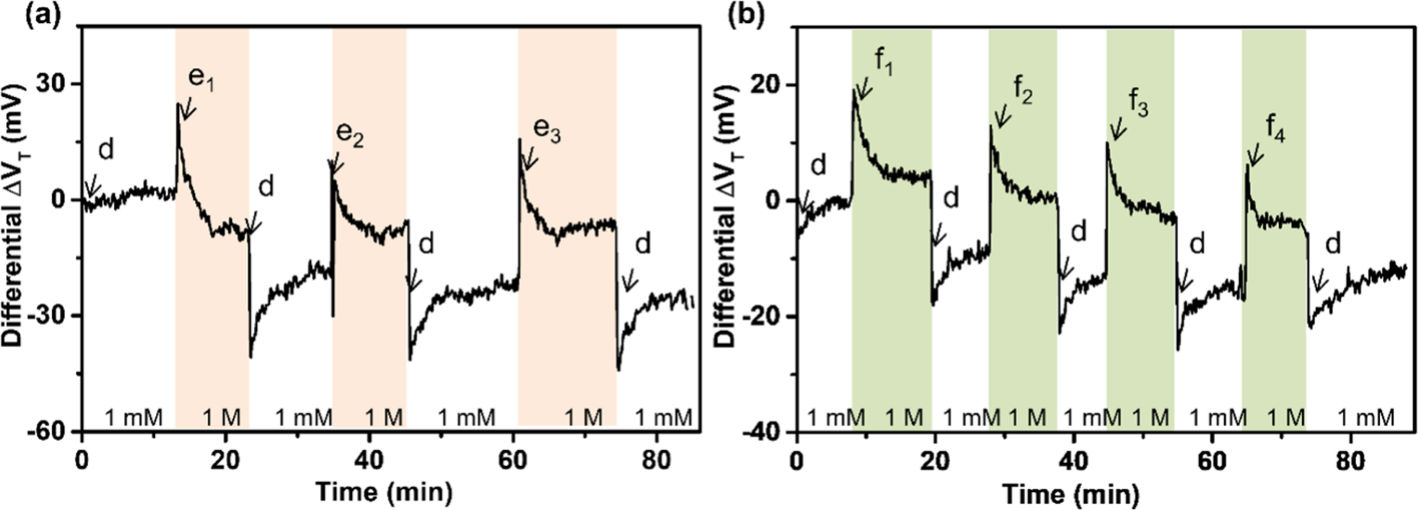
(a) Real-time potentiometric measurements showing the influence of exchanging the measurement and the hybridization buffer solutions. At point d, Tris buffer containing 1 mM NaCl was injected whereas at point e, Tris buffer containing 1 M NaCl was injected. The subscript numbers denote the different replications. (b) Real-time potentiometric measurements of DNA hybridization with the alternative method. These measurements were performed immediately after the experiment in (a). At point c, Tris buffer containing 1 mM NaCl was injected for 10 min whereas Tris buffers containing 1 mM NaCl and 1, 10, 100, or 1000 nM target DNA, respectively, were injected at the points f_1_, f_2_, f_3_, and f_4_. The flow rate for each injection was started from 100 μl/min for 10 s and was then kept at 5 μL/min.

As is evident from the literature, the requirements on the salt concentrations used in DNA hybridizations and charge registrations are contradictory.^19^ To increase the potentiometric signal due to the probe-target DNA hybridization, an alternative approach could be used to separate these two processes, using a high salt concentration for DNA hybridization and a low salt concentration for the charge registration.^15^ Our estimation based on SPR analysis suggests that such an alternative method, that is, hybridization in Tris buffer containing 1 M NaCl and charge registration in Tris buffer containing 1 mM NaCl, could significantly enhance the maximum change in the potentiometric signal from 44 mV (from diluting buffer method) to about 1 V.^15^ Here, it was assumed that the Γ_Target_ value was 2.9 × 10^12^ molecules/cm^2^ and that five bases from one target DNA were situated within λ_D_. To test the applicability of this modified approach, potentiometric DNA detection was therefore performed with the abovementioned SiNRFETs. Since different salt concentrations were used for the hybridization and charge registration, it is important to make sure that the solution exchange does not give rise to large potential changes. Therefore, the influence of the exchange between the measurement and hybridization buffers on the potentiometric signal was first investigated. As seen from the example in Figure 3a, Tris buffer containing 1 mM NaCl and Tris buffer containing 1 M NaCl were injected alternatively. The change in the differential Δ*V*_T_ value (measured in the 1 mM NaCl Tris buffer) before and after the injection of the 1 M NaCl Tris buffer gradually decreased with repeated injections. It was found that the differential Δ*V*_T_ change, caused by the solution exchange, was minimized after three to four repetitions (see Figure S2 for the results and an example of how to obtain the differential Δ*V*_T_).

Potentiometric detection of DNA was performed immediately after the initial influence of the 1 M NaCl injection had been eliminated. Real-time potentiometric responses after injecting 1, 10, 100, and 1000 nM target DNA (in 1 M NaCl) are shown in Figure 3b. The registered differential Δ*V*_T_ values in the 1 mM NaCl Tris buffer after the injections of these different concentrations of target DNA exhibit a small decreasing trend (Figure 3b). Similar results were also obtained when repeating the experiment three times on two different devices. Real-time experimental results for these repeated experiments and an example of the differential ΔV_T_ subtraction are shown in Figure S3. Since the DNA hybridization should result in a positive Δ*V*_T_, these small negative Δ*V*_T_ values suggest that no significant potentiometric signal, due to the surface DNA hybridization, was registered. This is puzzling, considering that the surface DNA hybridization was confirmed and quantified by SPR in our previous study.^15^

One plausible reason for the low DNA detection sensitivity could be that the potential of the gold electrode was not strictly linked to the double layer charging as assumed in our previous work,^15^ but to at least, one additional phenomenon acting as a buffer potential with respect to the charge induced by the surface DNA hybridization. In the presence of oxide on the sensing surface, the pH sensitivity on the oxide surface could buffer the change of the surface charge.^16−18^ However, as our sensing experiments were carried out in a pH 7.5 Tris buffer and our sample surface exhibited a quite low pH sensitivity (20 ± 2 mV/dec), the completely suppressed potentiometric signal for our DNA FETs cannot be explained by the pH sensitivity of the gold-sensing surface. On the other hand, the potential of a gold electrode can also be affected by redox species present in the electrolyte (such as oxygen) or on the gold surface itself.^20^

The surface of the gold-sensing electrode was modified by DNA/MCH via a self-assembly process involving the following thiol–gold reaction^21^
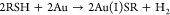
2where RSH and Au(I)SR denote the thiol in the solution and the thiol-Au(I) species formed on the Au surface, respectively. This surface functionalization process hence results in the formation of an Au(I)SR/Au redox couple on the sensing electrode surface. This Au(I)SR/Au redox couple should then give rise to a redox buffering effect. The redox reaction that yields the redox buffer capacity is
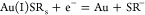
3

The potential of the Au(I)SR/Au-coated electrode [i.e., E(Au(I)SR/Au)] will then depend on the thiol concentration near the electrode surface (i.e., [SR^–^]) as shown in eq 4.
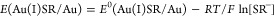
4where *E*^0^(Au(I)SR/Au) is the standard potential associated with eq 3, *R* is the ideal gas constant, *T* is the temperature, and *F* is the Faraday constant.

As seen in eq 4, the *E*(Au(I)SR/Au) will thus be determined by the [SR^–^] at the electrode surface. This should also be the case in our system based on the use of a probe DNA- and MCH-modified gold surface. Here, it should be mentioned that a thiolated self-assembled monolayer on gold is part of a dynamic equilibrium^22^ as has been shown via by the movement of etch pits,^23^ surface diffusion of the thiolates on the gold,^24^ and the exchange of thiols on the gold surface.^25^ This dynamic equilibrium should result in the establishment of a ratio between the concentration of the desorbed MCH and the MCH surface coverage density (Γ_MCH_). The surface potential of the probe DNA- and MCH-modified gold electrode may therefore become controlled by eq 4.

**Figure 4:**
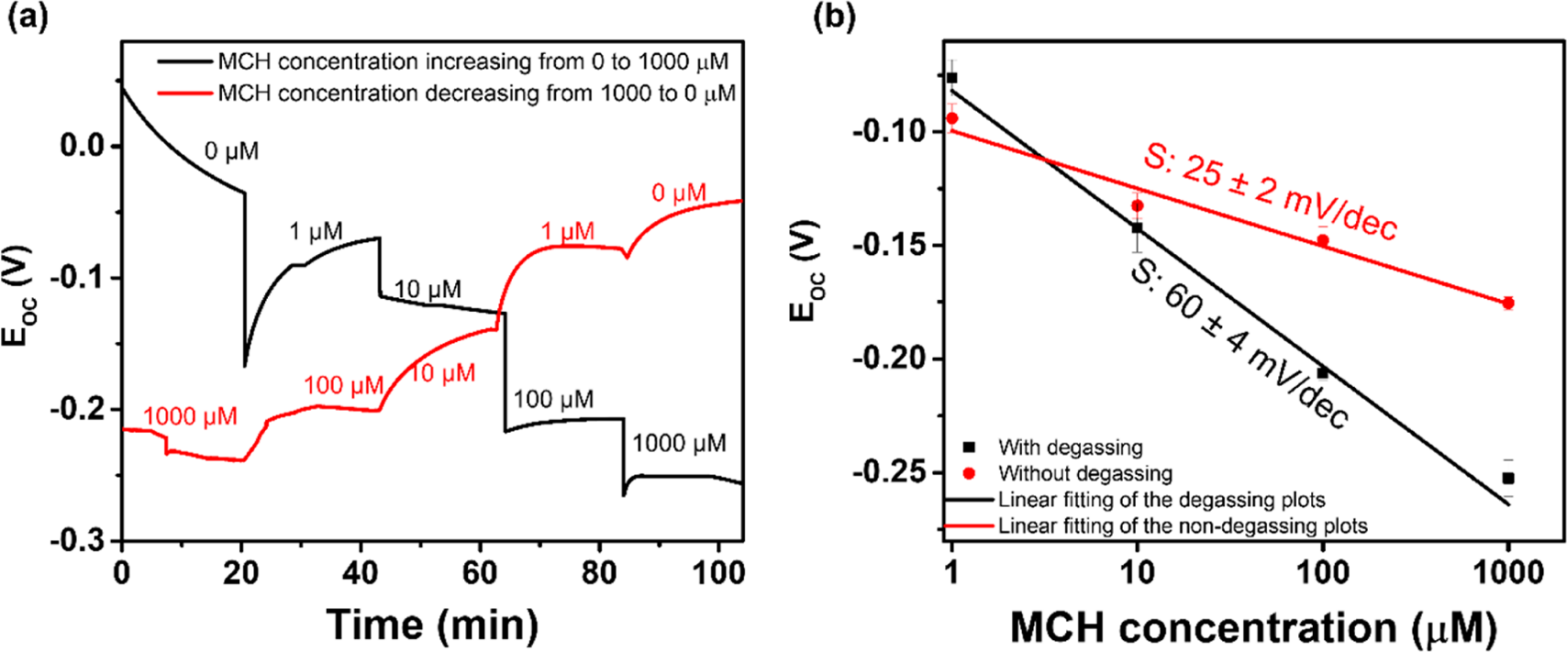
*E*_OC_ measurements to verify the redox buffering effects (a) *E*_OC_ of an MCH-modified gold electrode in the degassed electrolyte with varying concentrations of MCH. (b) *E*_OC_ vs MCH concentrations in the degassed and non-degassed electrolyte. The measurements were performed in a pH 7.5 Tris buffer containing 10 mM NaCl.

As the Au(I)SR/Au redox buffer hypothesis assumes that the Au(I)SR/Au is reversible enough to buffer the potential of the electrode during the DNA detection step, *E*_OC_ measurement experiments were carried out to examine whether the dependence of *E*(Au(I)SR/Au) on the MCH concentration in the electrolyte was in accordance with Nernst equation (see eq 4). Please note that the direction of the *E*_OC_ change of the electrode was opposite to the Δ*V*_T_ of the ISFET, since Δ*V*_T_ was compensating the potential change of the sensing electrode in the ISFET measurement. Moreover, it is well-known that MCH cannot form a perfect self-assembled monolayer (SAM) on the gold. The exposed gold sites induced by the SAM defects may also interact with other redox species, for example*,* O_2_, in the electrolyte at the same time as the occurrence of the gold–thiol redox reaction. To avoid complications induced by oxygen, these experiments were first performed in a degassed electrolyte. As seen in Figure 4a, the *E*_OC_ of the MCH-modified Au electrode decreased with the MCH concentration increasing regardless of whether the MCH concentration was manipulated upward or downward. The obtained slope of the *E*_OC_ versus MCH concentration in the degassed electrolyte was 60 ± 4 mV/dec where the error bar depicts the standard deviations based on four independent experiments (see Figure 4b). The 60 ± 4 mV/dec slope in the degassed electrolyte is not significantly different from the Nernstian response, which confirms that the Au(I)SR/Au system is sufficiently reversible to control the electrode potential in the absence of oxygen.

Since the potentiometric DNA detection experiments were performed in the non-degassed electrolyte, the influence of oxygen on the *E*_OC_ of the MCH-modified Au electrode for different concentrations of MCH was also investigated. Examples of *E*_OC_ versus t curves for different MCH concentrations in the degassed and non-degassed electrolytes can be found in Figure S4. The slope of the *E*_OC_ versus MCH concentration plot obtained in the non-degassed electrolyte (Figure 4b, red line) was 25 ± 2 mV/dec with the error bar again depicting the standard deviations based on four independent experiments. The difference between the slopes for the degassed and non-degassed electrolyte demonstrated that the dissolved oxygen also influenced the potential of the MCH-modified gold surface. The potential of the MCH-modified gold surface is therefore determined by the mixed potential of the gold–thiol and the gold–oxygen reactions. The possible gold–oxygen reactions could be the oxygen oxidizing Au to Au(I) and the oxygen reduction reaction. In addition, it is worth noting that the mixed potential can be achieved by different combinations of gold–oxygen and gold–thiol redox reactions depending on the reaction kinetics. The requisite for the combinations is the reduction current is equal to the oxidation current.

A rough estimation may facilitate the understanding of the influence of this redox buffer effect. In this estimation, we assume that the immobilization of the probe DNA did not change Γ_MCH_ and that Γ_MCH_ was equal to 2.75 × 10^–9^ mol/cm^2^ according to the previous findings.^26^ It is also assumed that the target DNA was hybridized with the probe DNA (Γ_Probe_ = 6.8 × 10^12^ molecules/cm^2^, equal to 1.1 × 10^–11^ mol/cm^2^) with an efficiency of 100% and that five DNA bases on one target DNA were within λ_D_. In this extreme case, the negative charge induced by the hybridized target DNA would be about 5.6 × 10^–11^ mol/cm^2^. To buffer this potential change, due to the additional negative charge on the electrode, only 1% of the MCH would need to be reductively desorbed from the sensing surface which hence would leave 99% of the MCH on the sensing surface. The resulting potential change of such a limited loss of Au(I)SR from the electrode surface would then be limited to about 0.0.3 mV. Such a small potential change would clearly not be detectable under the present experimental conditions. It should, however, also be noted that the potential change should be even smaller in a real experiment as Γ_Target_ should be significantly lower than the value used in the estimation due to steric hindrance.^15,19^

Our results consequently indicate that the failure to detect the surface charge change caused by the DNA hybridization is due to the redox buffering effect caused by the mixed potential of the gold–thiol and gold–oxygen redox interactions. The same effect could also apply for other potentiometric detections of biomarkers based on the thiol–gold chemistry.

## Conclusions

Potentiometric DNA sensing with a gold-sensing electrode was carefully investigated using a SiNRFET sensor. It was found that the Au(I)SR/Au redox couple, introduced via the thiol-based functionalization process can fix the potential of the thiolated gold-sensing electrode together with the oxygen effect. The mixed potential of the gold–thiol and gold–oxygen redox interactions can buffer the incoming charge induced by the surface DNA hybridization, thus suppressing the potentiometric signal. The same effect could also apply for other potentiometric detections of biomarkers using thiol–gold chemistry.

## Supporting Information Available

All the potentiometric DNA detection results and comparison of *E*_OC_ versus *t* curves with MCH concentrations change in the degassed and non-degassed electrolytes (PDF)

## References

[ref1] Bergveld P. Thirty Years of ISFETOLOGY: What Happened in the Past 30 Years and What May Happen in the next 30 Years. Sens. Actuators, B 2003, 88, 1–20. 10.1016/s0925-4005(02)00301-5.

[ref2] Cui Y.; Lieber C. M. Functional Nanoscale Electronic Devices Assembled Using Silicon Nanowire Building Blocks. Science 2001, 291, 851–853. 10.1126/science.291.5505.851. 11157160

[ref3] Lee C.-S.; Kim S.; Kim M. Ion-Sensitive Field-Effect Transistor for Biological Sensing. Sensors 2009, 9, 7111–7131. 10.3390/s90907111. 22423205 PMC3290489

[ref4] Wipf M.; Stoop R. L.; Tarasov A.; Bedner K.; Fu W.; Wright I. A.; Martin C. J.; Constable E. C.; Calame M.; Schönenberger C. Selective Sodium Sensing with Gold-Coated Silicon Nanowire Field-Effect Transistors in a Differential Setup. ACS Nano 2013, 7, 5978–5983. 10.1021/nn401678u. 23768238

[ref5] Fritz J.; Cooper E. B.; Gaudet S.; Sorger P. K.; Manalis S. R. Electronic Detection of DNA by Its Intrinsic Molecular Charge. Proc. Natl. Acad. Sci. U.S.A. 2002, 99, 14142–14146. 10.1073/pnas.232276699. 12386345 PMC137851

[ref6] Sorgenfrei S.; Chiu C.-y.; Gonzalez R. L.; Yu Y.-J.; Kim P.; Nuckolls C.; Shepard K. L. Label-Free Single-Molecule Detection of DNA-Hybridization Kinetics with a Carbon Nanotube Field-Effect Transistor. Nat. Nanotechnol. 2011, 6, 126–132. 10.1038/nnano.2010.275. 21258331 PMC3783941

[ref7] Gao A.; Lu N.; Wang Y.; Dai P.; Li T.; Gao X.; Wang Y.; Fan C. Enhanced Sensing of Nucleic Acids with Silicon Nanowire Field Effect Transistor Biosensors. Nano Lett. 2012, 12, 5262–5268. 10.1021/nl302476h. 22985088

[ref8] Rani D.; Pachauri V.; Ingebrandt S. Silicon Nanowire Field-Effect Biosensors. In Label-Free Biosensing: Advanced Materials, Devices and Applications; Schöning M. J., Poghossian A., Eds.; Springer International Publishing: Cham, 2018, pp 27–57. Springer Series on Chemical Sensors and Biosensors 10.1007/5346_2017_19.

[ref9] Ishige Y.; Shimoda M.; Kamahori M. Immobilization of DNA Probes onto Gold Surface and Its Application to Fully Electric Detection of DNA Hybridization Using Field-Effect Transistor Sensor. Jpn. J. Appl. Phys. 2006, 45, 3776 10.1143/jjap.45.3776.

[ref10] Sakata T.; Matsumoto S.; Nakajima Y.; Miyahara Y. Potential Behavior of Biochemically Modified Gold Electrode for Extended-Gate Field-Effect Transistor. Jpn. J. Appl. Phys. 2005, 44, 2860 10.1143/jjap.44.2860.

[ref11] Poghossian A.; Cherstvy A.; Ingebrandt S.; Offenhäusser A.; Schöning M. J. Possibilities and Limitations of Label-Free Detection of DNA Hybridization with Field-Effect-Based Devices. Sens. Actuators, B 2005, 111-112, 470–480. 10.1016/j.snb.2005.03.083.

[ref12] Cherstvy A. G. Detection of DNA Hybridization by Field-Effect DNA-Based Biosensors: Mechanisms of Signal Generation and Open Questions. Biosens. Bioelectron. 2013, 46, 162–170. 10.1016/j.bios.2013.02.026. 23542899

[ref13] Kalra S.; Kumar M. J.; Dhawan A. Dielectric-Modulated Field Effect Transistors for DNA Detection: Impact of DNA Orientation. IEEE Electron Device Lett. 2016, 37, 1485–1488. 10.1109/led.2016.2613110.

[ref14] Bunimovich Y. L.; Shin Y. S.; Yeo W.-S.; Amori M.; Kwong G.; Heath J. R. Quantitative Real-Time Measurements of DNA Hybridization with Alkylated Nonoxidized Silicon Nanowires in Electrolyte Solution. J. Am. Chem. Soc. 2006, 128, 16323–16331. 10.1021/ja065923u. 17165787 PMC3695614

[ref15] Xu X.; Makaraviciute A.; Abdurakhmanov E.; Wermeling F.; Li S.; Danielson U. H.; Nyholm L.; Zhang Z. Estimating Detection Limits of Potentiometric DNA Sensors Using Surface Plasmon Resonance Analyses. ACS Sens. 2020, 5, 217–224. 10.1021/acssensors.9b02086. 31833355

[ref16] Landheer D.; McKinnon W. R.; Aers G.; Jiang W.; Deen M. J.; Shinwari M. W. Calculation of the Response of Field-Effect Transistors to Charged Biological Molecules. IEEE Sens. J. 2007, 7, 1233–1242. 10.1109/jsen.2007.901047.

[ref17] Schasfoort R. B. M.; Bergveld P.; Kooyman R. P. H.; Greve J. Possibilities and Limitations of Direct Detection of Protein Charges by Means of an Immunological Field-Effect Transistor. Anal. Chim. Acta 1990, 238, 323–329. 10.1016/s0003-2670(00)80554-1.

[ref18] Stoop R. L.; Wipf M.; Müller S.; Bedner K.; Wright I. A.; Martin C. J.; Constable E. C.; Fu W.; Tarasov A.; Calame M.; Schönenberger C. Competing Surface Reactions Limiting the Performance of Ion-Sensitive Field-Effect Transistors. Sens. Actuators, B 2015, 220, 500–507. 10.1016/j.snb.2015.05.096.

[ref19] Halperin A.; Buhot A.; Zhulina E. B. On the Hybridization Isotherms of DNA Microarrays: The Langmuir Model and Its Extensions. J. Phys.: Condens. Matter 2006, 18, S463 10.1088/0953-8984/18/18/s01.

[ref20] Bard A. J.; Faulkner L. R.; Leddy J.; Zoski C. G. Electrochemical Methods: Fundamentals and Applications; Wiley: New York, 1980; Vol. 2.

[ref21] Vericat C.; Vela M. E.; Benitez G.; Carro P.; Salvarezza R. C.; Salvarezza C. R. Self-assembled monolayers of thiols and dithiols on gold: new challenges for a well-known system. Chem. Soc. Rev. 2010, 39, 1805–1834. 10.1039/b907301a. 20419220

[ref22] Bürgi T. Properties of the gold-sulphur interface: from self-assembled monolayers to clusters. Nanoscale 2015, 7, 15553–15567. 10.1039/c5nr03497c. 26360607

[ref23] Poirier G. E.; Tarlov M. J. Molecular Ordering and Gold Migration Observed in Butanethiol Self-Assembled Monolayers Using Scanning Tunneling Microscopy. J. Phys. Chem. 1995, 99, 10966–10970. 10.1021/j100027a042.

[ref24] Stranick S. J.; Parikh A. N.; Allara D. L.; Weiss P. S. A New Mechanism for Surface Diffusion: Motion of a Substrate-Adsorbate Complex. J. Phys. Chem. 1994, 98, 11136–11142. 10.1021/j100094a024.

[ref25] Leung K. K.; Gaxiola A. D.; Yu H.-Z.; Bizzotto D. Tailoring the DNA SAM Surface Density on Different Surface Crystallographic Features Using Potential Assisted Thiol Exchange. Electrochim. Acta 2018, 261, 188–197. 10.1016/j.electacta.2017.12.114.

[ref26] Makaraviciute A.; Xu X.; Nyholm L.; Zhang Z. Systematic Approach to the Development of Microfabricated Biosensors: Relationship between Gold Surface Pretreatment and Thiolated Molecule Binding. ACS Appl. Mater. Interfaces 2017, 9, 26610–26621. 10.1021/acsami.7b08581. 28726367

