# Supplementary material for: Redox Buffering Effects in Potentiometric Detection of DNA Using Thiol-Modified Gold Electrodes
Source: ACS Sens. 2021 Jun 29;6(7):2546–52. doi: 10.1021/acssensors.0c02700 (PMC8314270; doi:10.1021/acssensors.0c02700)
Supplement: Supplementary file 1 — se0c02700_si_001.pdf [file se0c02700_si_001.pdf]

Redox Buffering Effects in Potentiometric Detection of DNA Using Thiol modified Gold Electrodes

Xingxing Xu[1], Yingtao Yu[1], Qitao Hu[1], Si Chen[1], Leif Nyholm[2], Zhen Zhang[1]*

AUTHOR ADDRESS

[1]Division of Solid-State Electronics, Department of Electrical Engineering, Ångström Laboratory, Uppsala University, P.O. Box 65, SE-75103, Uppsala, Sweden

[2]Department of Chemistry-Ångström Laboratory, Uppsala University, P.O. Box 538, SE-751 21 Uppsala, Sweden

*Corresponding author: Zhen.Zhang@angstrom.uu.se

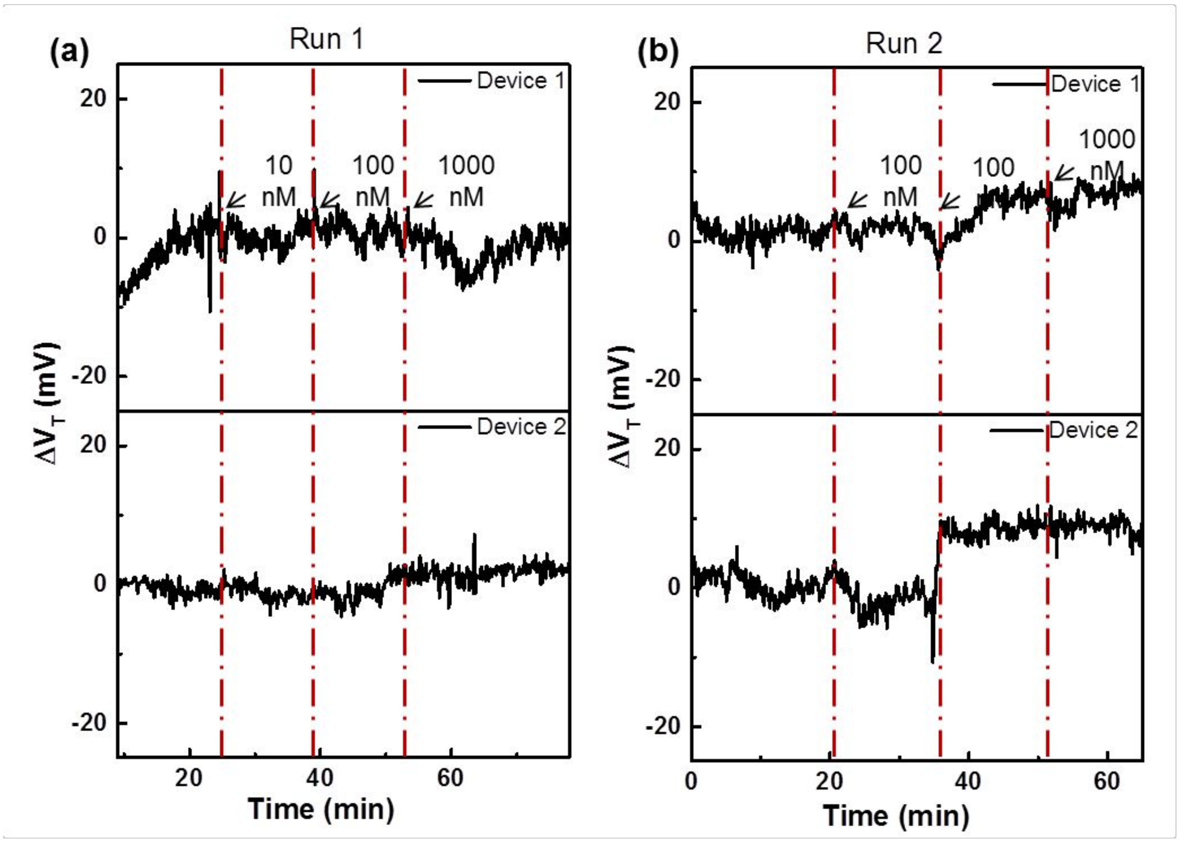

Figure S1. Real-time potentiometric detection of DNA in the tris buffer containing 10 mM NaCl on the 2 different sensing SiNRFETs (a) run 1; (b) run 2. The injected target concentration was 10 nM, 100 nM and 1000 nM, respectively. The flow rate was 5 μl/min and the time interval between each injection was around 15 minutes.

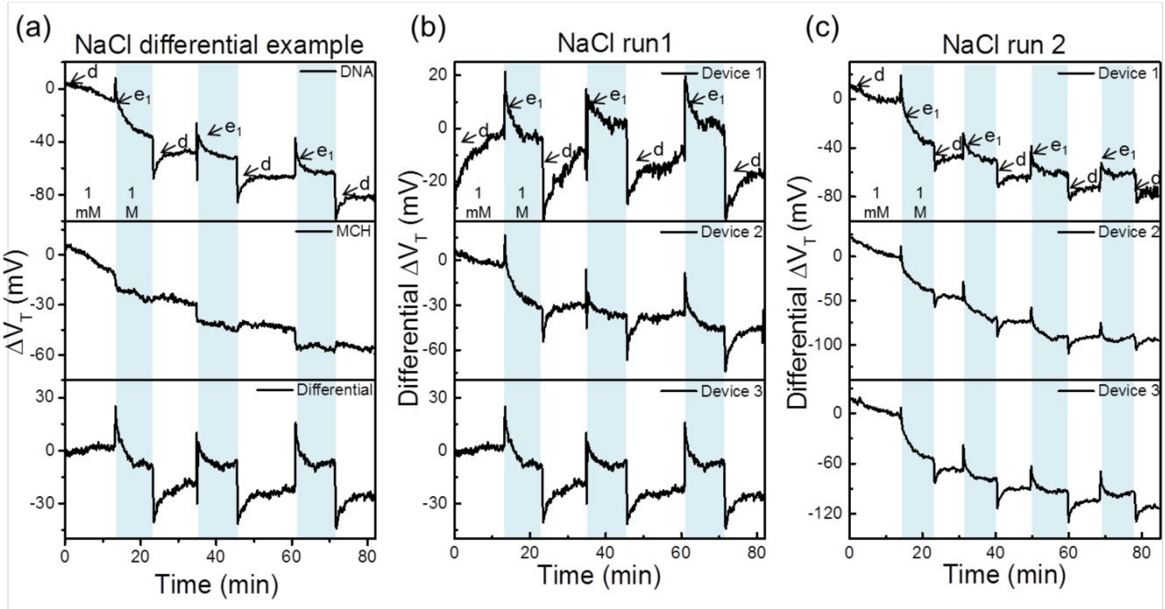

Figure S2. Real-time potentiometric measurement showing the influence of exchanging the measurement and the hybridization buffer solutions. (a) An example showing how the differential ΔV_T was extracted. (b) and (c) The results for three devices and two repeating experiments. At point d, the tris buffer containing 1 mM NaCl was injected; at point e, the tris buffer containing 1 M NaCl was injected, the subscript numbers represent different replications.

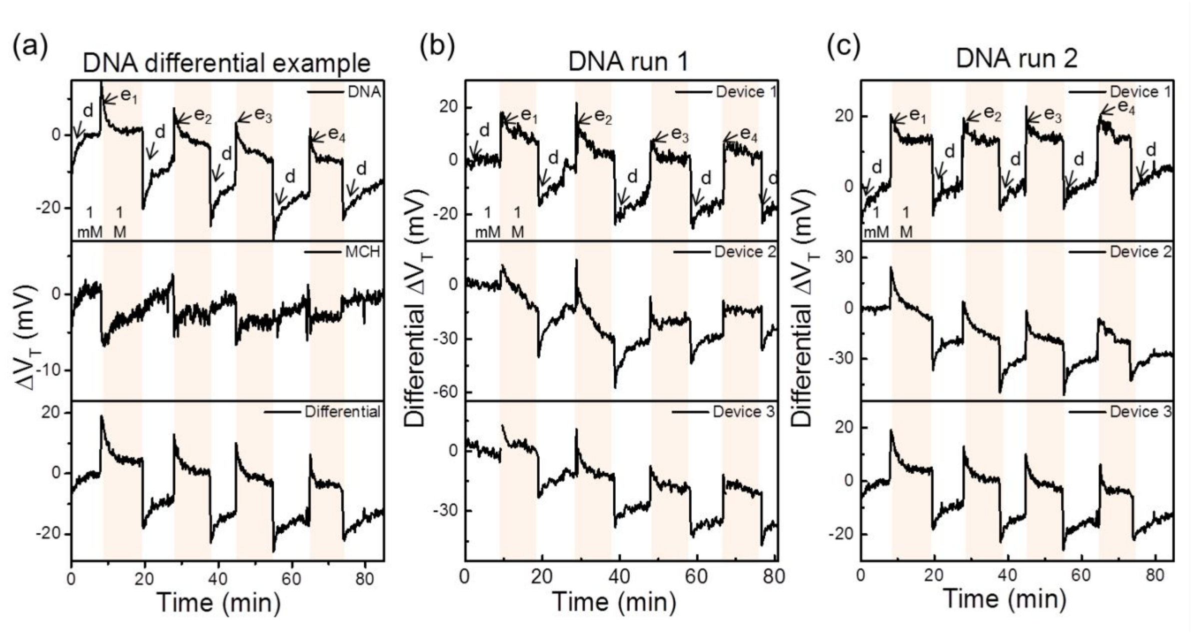

Figure S3. Real-time potentiometric measurement of DNA hybridization with the alternative method. This measurement was performed immediately after the experiment in Figure S2. At point d, the tris buffer containing 1 mM NaCl was injected for 10 minutes; at point e, the tris buffer containing 1 mM NaCl and different concentrations of target DNA was injected where e_1, e_2, 3_3 and e_4 represent 1, 10, 100, and 1000 nM target DNA, respectively. The flow rate was 5 µl/min.

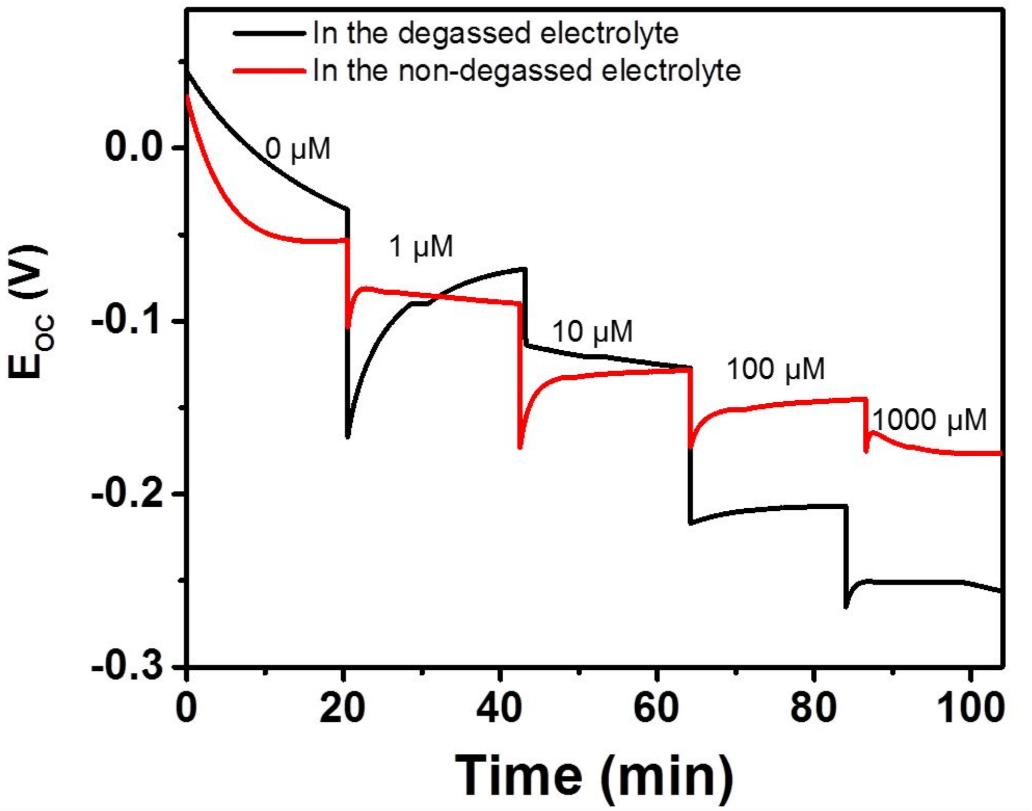

Figure S4. The comparison of the E_{OC} vs. t with changing MCH concentrations in the degassed (black) and non-degassed (red) electrolyte. The supporting electrolyte is 10 mM tris-HCl containing 10 mM NaCl.
